# Supplementary material for: Efficient generation of a self-organizing neuromuscular junction model from human pluripotent stem cells
Source: Nat Commun. 2023 Dec 19;14:8043. doi: 10.1038/s41467-023-43781-3 (PMC10730704; doi:10.1038/s41467-023-43781-3)
Supplement: Supplementary file 1 — Supplementary Information [file 41467_2023_43781_MOESM1_ESM.pdf]

Supplementary Information

Efficient generation of a self-organizing neuromuscular junction model from human pluripotent stem cells

Alessia Urzi[1], Ines Lahmann[#1], Lan Vi Ngoc Nguyen[#1], Benjamin R. Rost[#2], Angelica Garcia Perez[1], Noemie Lelievre[1], Megan E. Merritt-Garza[3], Han C. Phan[4], Gary J. Bassell[3], Wilfried Rossoll[5], Sebastian Diecke[6], Severine Kunz[7], Dietmar Schmitz[2,8], Mina Gouti[1]

1. Stem Cell Modelling of Development & Disease Group, Max Delbrück Center for Molecular Medicine in the Helmholtz Association (MDC), 13125 Berlin, Germany;
2. German Center for Neurodegenerative Diseases (DZNE), Berlin, Germany
3. Department of Cell Biology, Laboratory for Translational Cell Biology, Emory University School of Medicine, Atlanta, GA 30322, USA
4. Department of Pediatrics, University of Alabama, Birmingham, AL 35294, USA;
5. Department of Neuroscience, Mayo Clinic, Jacksonville, FL 32224, USA; Mayo Clinic Graduate School of Biomedical Sciences, Mayo Clinic, Jacksonville, FL 32224, USA.
6. Max-Delbrück-Center for Molecular Medicine in the Helmholtz Association (MDC), Technology Platform Pluripotent Stem Cells, 13125 Berlin, Germany
7. Max-Delbrück-Center for Molecular Medicine in the Helmholtz Association (MDC), Technology Platform Electron Microscopy, 13125 Berlin, Germany.
8. Berlin Institute of Health, NeuroCure Cluster of Excellence, Charité-Universitätsmedizin Berlin, Corporate Member of Freie Universität Berlin and Humboldt-Universität zu Berlin, Berlin, Germany.

# These authors contributed equally.

Corresponding author: mina.gouti@mdc-berlin.de

Supplementary Figure S1

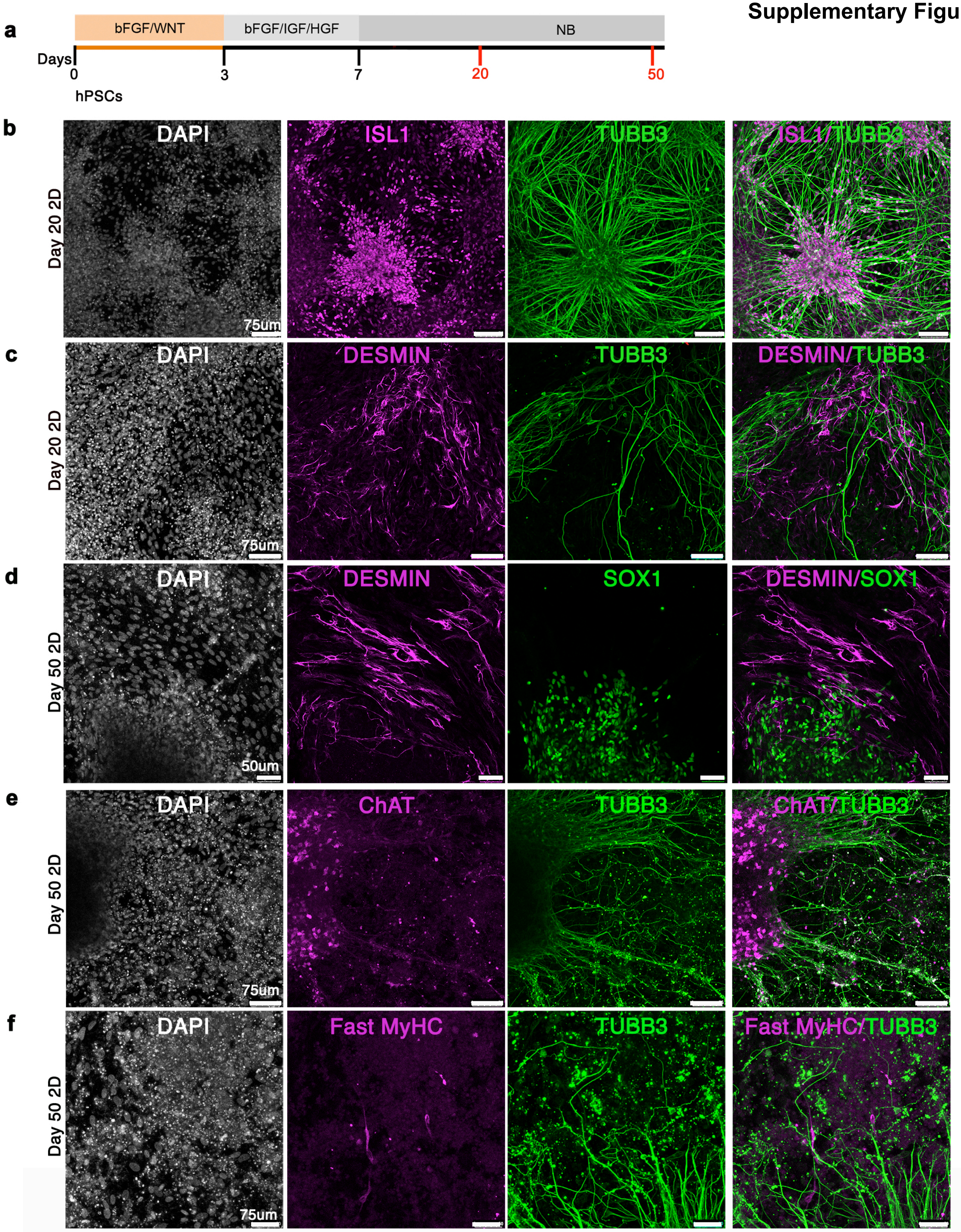

Supplementary Figure S1: Differentiation of hPSCs by adapting the NMO protocol to adherent culture conditions is not sufficient to generate NMJs.

(a) Differentiation strategy adapted from the NMO protocol to adherent culture conditions.

(b-c) Immunofluorescence analysis at day 20 revealed the presence of MNs expressing ISL1/TUBB3 and myoblasts expressing DESMIN. Scale bars 75 μm.

(d-f) Immunofluorescence analysis at day 50 showed that DESMIN^{+} myoblasts and SOX1^{+} NPs were still present in the culture. The MNs expressed ChAT but the myoblasts failed to differentiate to mature muscle fibers expressing Fast MyHC. Scale bars 50 μm (d) and 75 μm (e,f).

hPSCs: human pluripotent stem cells; NMO: neuromuscular organoid; NMJs: neuromuscular junctions; MNs: motor neurons; NPs: neural progenitors.

Supplementary Figure S2

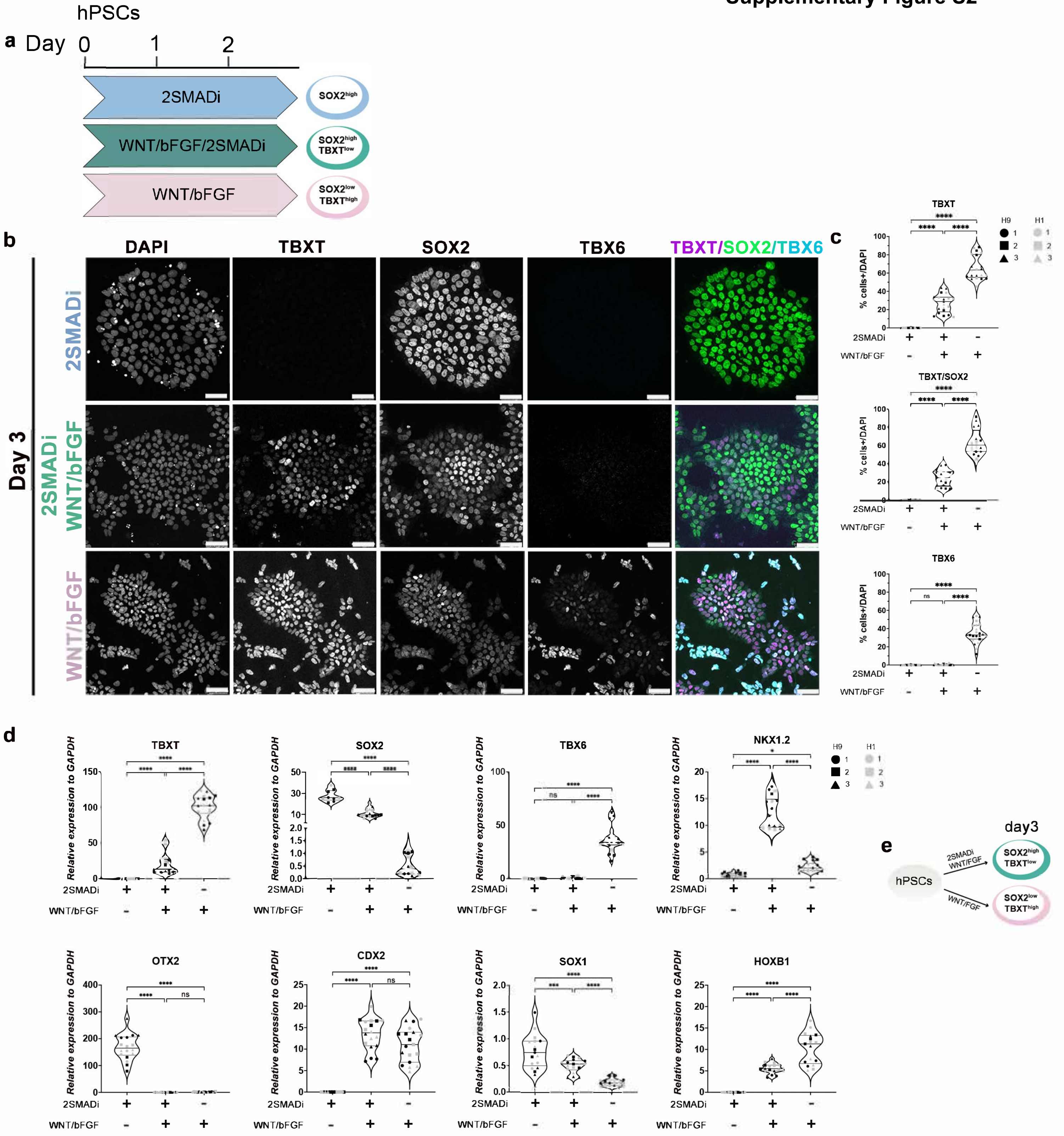

Supplementary Figure S2: Effect of 2SMAD inhibition on the differentiation of hPSC derived NMPs.

(a) Schematic illustration of hPSCs differentiation strategy.

(b) 2SMADi inhibition from day 0 – 3 resulted in the generation of anterior neural progenitor cells expressing SOX2. Simultaneous exposure of hPSCs to CHIR/bFGF and 2SMADi resulted in the generation of NMP-like cells that express high levels of SOX2 and low levels of TBXT in the absence of TBX6. Treatment of hPSCs with CHIR/bFGF from day 0 – 3 directed their differentiation to NMP cells co-expressing TBXT/SOX2 surrounded by TBX6^{+} cells. Scale bar 50 µm.

(c) Quantifications of TBXT, TBXT/SOX2, and TBX6 expression showing that 65.6% ± 12.9% of NMPs expressed TBXT, 64.2% ± 13.8% co-expressed TBXT/SOX2, and 35% ± 10.8% expressed TBX6. When exposed to 2SMADi and WNT/bFGF since day 0, 26.9% ± 10.1% of the cells expressed TBXT and 24.4% ± 8.9% co-expressed TBXT/SOX2, while TBX6 was downregulated (0.26% ± 0.6%) (H1: N=3, n=9; H9: N=3, n=9). The statistical tests employed included an unpaired t-test with Welch's correction. *P ≤ 0.05; **P ≤0.01; ***P ≤ 0.001; ****P ≤ 0.0001. Each dot with different colour represents different PSC lines, and different shapes represent different experiments. Source data are provided as a Source Data file.

(d) qPCR analysis of day 3 cells treated from day 0 - 3 with 2SMADi alone or with CHIR/bFGF and 2SMADi, or CHIR/bFGF alone. Cells treated with 2SMADi alone expressed the anterior marker *OTX2* and the NP markers *SOX2* and *SOX1*. The combination of 2SMADi with CHIR/bFGF resulted in the generation of NMP-like cells expressing low levels of *TBXT* and high levels of *SOX2* in the absence of *TBX6*. Exposure to CHIR/bFGF alone resulted in the generation of NMP cells expressing high levels of TBXT and low levels of SOX2 associated with an NMP identity. CDX2 associated with a posterior identity was expressed both in 2SMADi/CHIR/bFGF and CHIR/bFGF conditions (H1: N=3, n=9; H9: N=3, n=9). The statistical tests employed included an unpaired t-test with Welch's correction. *P ≤ 0.05; **P ≤0.01; ***P ≤ 0.001; ****P ≤ 0.0001. Each dot with different colour represents different PSC lines, and different shapes represent different experiments. Source data are provided as a Source Data file.

(e) Schematic illustration summarizing the levels of SOX2 and TBXT expression after treatment of hPSCs from day 0 - 3 with WNT/bFGF/2SMADi or WNT/bFGF alone.

hPSCs: human pluripotent stem cells; 2SMADi: dual-SMAD-inhibition; NMPs: neuromesodermal progenitors; ANPs: anterior neural progenitors.

Supplementary Figure S3

a Motor neurons progenitors - soNMJ model

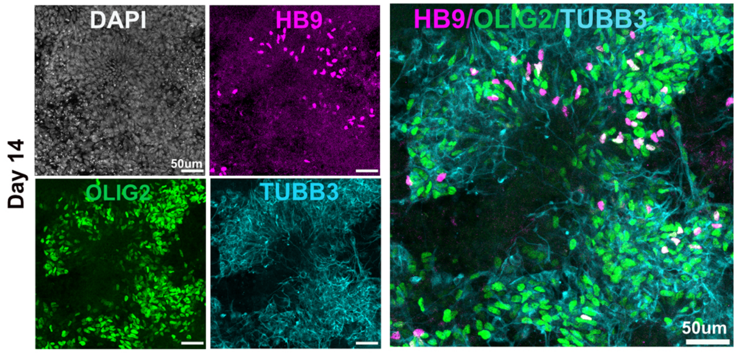

b

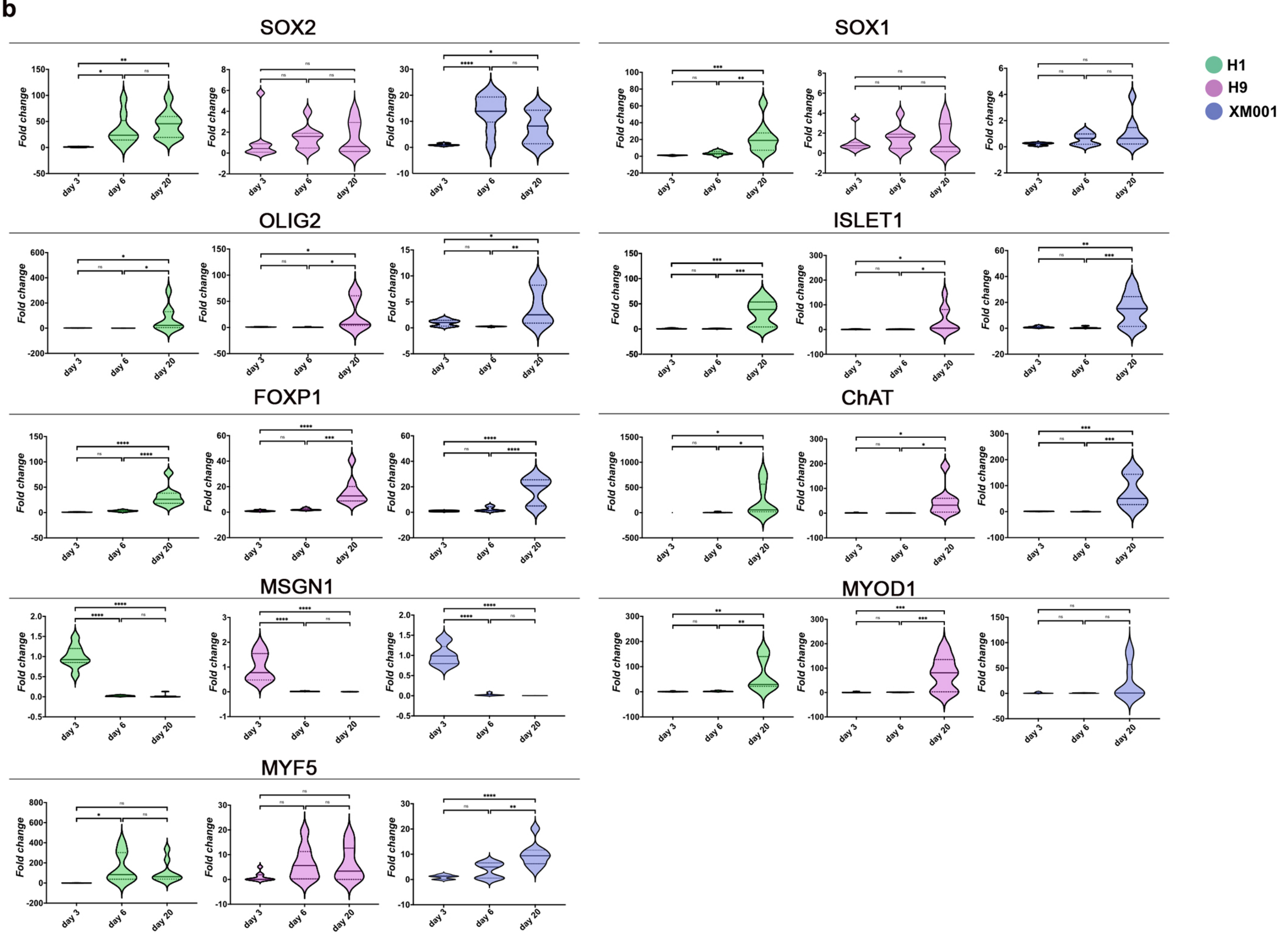

Supplementary Figure S3: Progressive differentiation of NMP cells to spinal cord neurons and muscle cells.

(a) Immunofluorescence analysis at day 14 showed the presence of $OLIG2^{+}$ MN progenitors and $HB9^{+}$ post-mitotic MNs. Scale bar 50µm.

(b) qPCR expression analysis at different developmental stages corresponding to day 3, 6 and 20 revealed the progressive differentiation of NMPs to NPs (SOX2, SOX1, OLIG2), mature MNs (ISL1, FOXP1, ChAT) and skeletal muscle progenitors (MYF5, MYOD). Fold change is determined in all conditions compared to day 3. The early mesodermal marker MSGN1 was downregulated. (H1: N=3, n=9; H9: N=3, n=9; XM001 N=3, n=9). The statistical tests employed included an unpaired t-test with Welch's correction. *P ≤ 0.05; **P ≤0.01; ***P ≤ 0.001; ****P ≤ 0.0001. Violin plots with different colours represent different PSC lines and different shapes represent different experiments. Source data are provided as a Source Data file.

MN: motor neuron; NMPs: neuromesodermal progenitors; NPs: neural progenitors.

Supplementary Figure S4

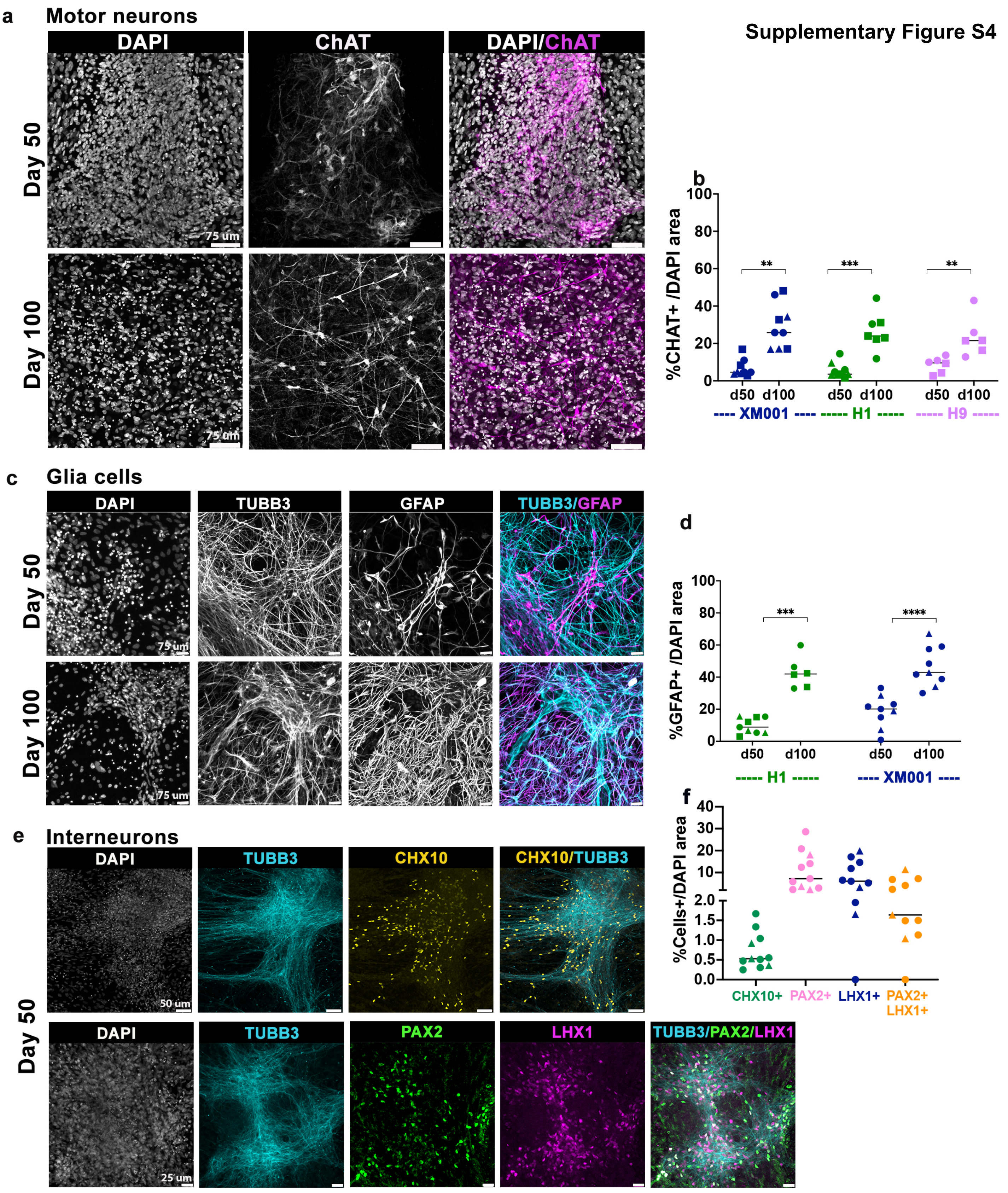

Supplementary Figure S4: Presence of motor neurons, glia cells and interneurons in soNMJ cultures.

(a) Immunofluorescence analysis of the soNMJ cultures at day 50 and 100 reveals the presence of $CHAT^+$ MNs. Scale bar 75µm.

(b) Quantification of $CHAT^+$ area / DAPI area at day 50 and 100 (H1: N=2-3, n=3-9; H9: N=2, n=6; XM001: N=3, n=9). Statistical analysis included an ordinary two-way ANOVA test. *P ≤ 0.05; **P ≤0.01; ***P ≤ 0.001; ****P ≤ 0.0001. Source data are provided as a Source Data file.

(c) Immunofluorescence analysis of the soNMJ cultures at day 50 and 100 reveals the presence of $GFAP^+$ glia cells. Scale bar 75 µm.

(d) Quantification of $GFAP^+$ area / DAPI area at day 50 and 100 (H1: N=2-3, n=6-9; XM001: N=3, n=9). The statistical tests employed included an unpaired t-test with Welch's correction. *P ≤ 0.05; **P ≤0.01; ***P ≤ 0.001; ****P ≤ 0.0001. Source data are provided as a Source Data file.

(e) At day 50, V2a interneurons were identified in the soNMJ model by immunofluorescence analysis of CHX10 and TUBB3 expression. V1 interneurons were identified by the co-expression of PAX2, LHX1, and TUBB3. Scale bar 25 µm and 50 µm.

(f) Quantification of $CHX10^+$, $PAX2^+$, $LHX1^+$ and $PAX2^+LHX1^+$ area / DAPI area at day 50 (H1: N=2, n=11). Source data are provided as a Source Data file.

Supplementary Figure S5

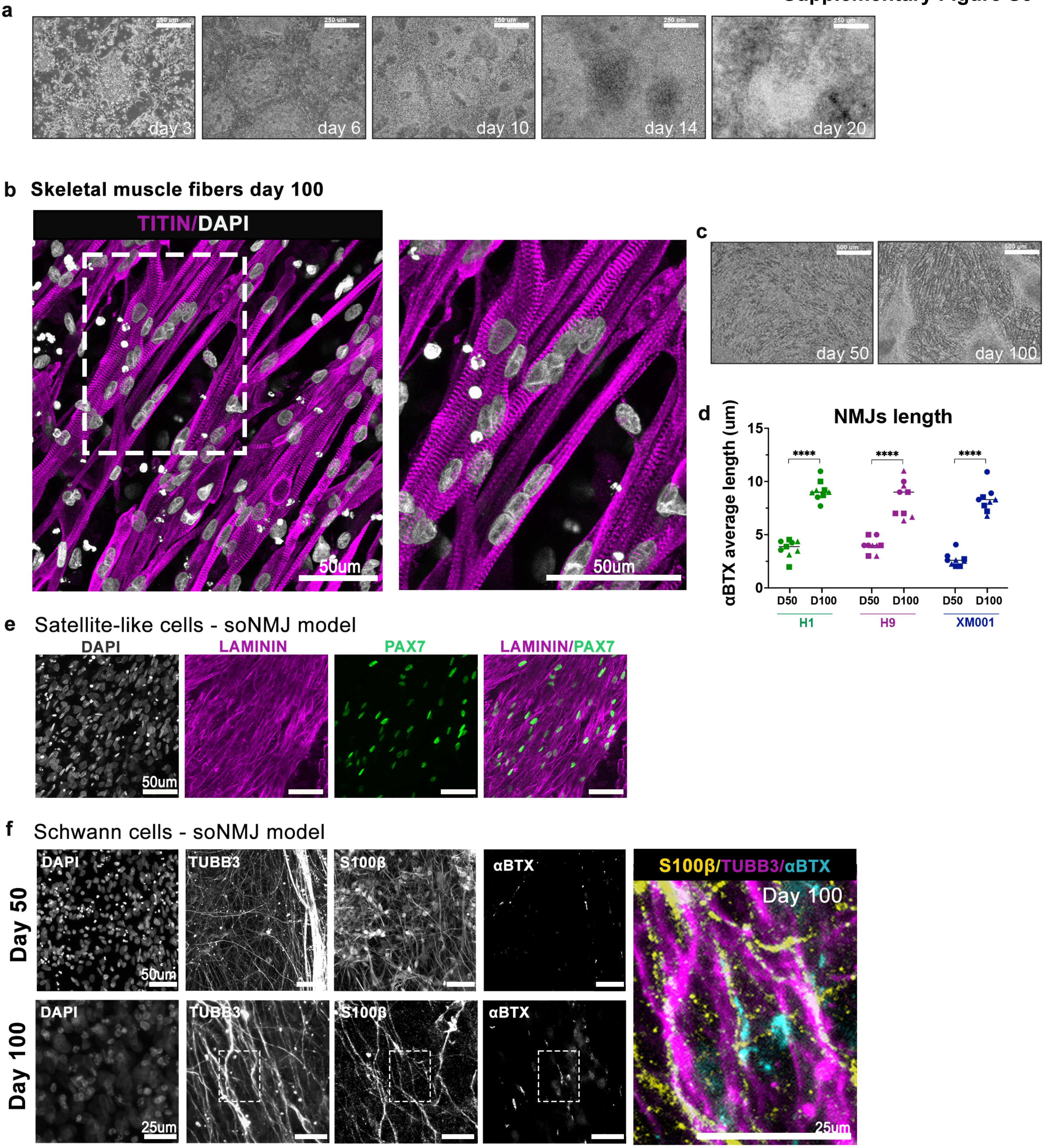

Supplementary Figure S5: Presence of motor neurons, glia cells and interneurons in soNMJ cultures.

(a) Immunofluorescence analysis of the soNMJ cultures at day 50 and 100 reveals the presence of $CHAT^+$ MNs. Scale bar 75µm.

(b) Quantification of $CHAT^+$ area / DAPI area at day 50 and 100 (H1 d50: N=3, n=9; H1 d100 N=3, n=7; H9: N=2, n=6; XM001: N=3, n=9). Statistical analysis included an ordinary two-way ANOVA test. *P ≤ 0.05; **P ≤0.01; ***P ≤ 0.001; ****P ≤ 0.0001. Different colours represent different PSC lines and different shapes represent different experiments. Source data are provided as a Source Data file.

(c) Immunofluorescence analysis of the soNMJ cultures at day 50 and 100 reveals the presence of $GFAP^+$ glia cells. Scale bar 75 µm.

(d) Quantification of $GFAP^+$ area / DAPI area at day 50 and 100 (H1 d50: N=3, n=9; H1 d100: N=2, n=6; XM001: N=3, n=9). The statistical tests employed included an unpaired t-test with Welch's correction. *P ≤ 0.05; **P ≤0.01; ***P ≤ 0.001; ****P ≤ 0.0001. Different colours represent different PSC lines and different shapes represent different experiments. Source data are provided as a Source Data file.

(e) At day 50, V2a interneurons were identified in the soNMJ model by immunofluorescence analysis of CHX10 and TUBB3 expression. V1 interneurons were identified by the co-expression of PAX2, LHX1, and TUBB3. Scale bar 25 µm and 50 µm.

(f) Quantification of $CHX10^+$, $PAX2^+$, $LHX1^+$ and $PAX2^+LHX1^+$ area / DAPI area at day 50 (H1: N=2, n=11). Different shapes represent different experiments. Source data are provided as a Source Data file.

soNMJ: self-organizing neuromuscular junction; PSC: pluripotent stem cells.

Supplementary Figure S6

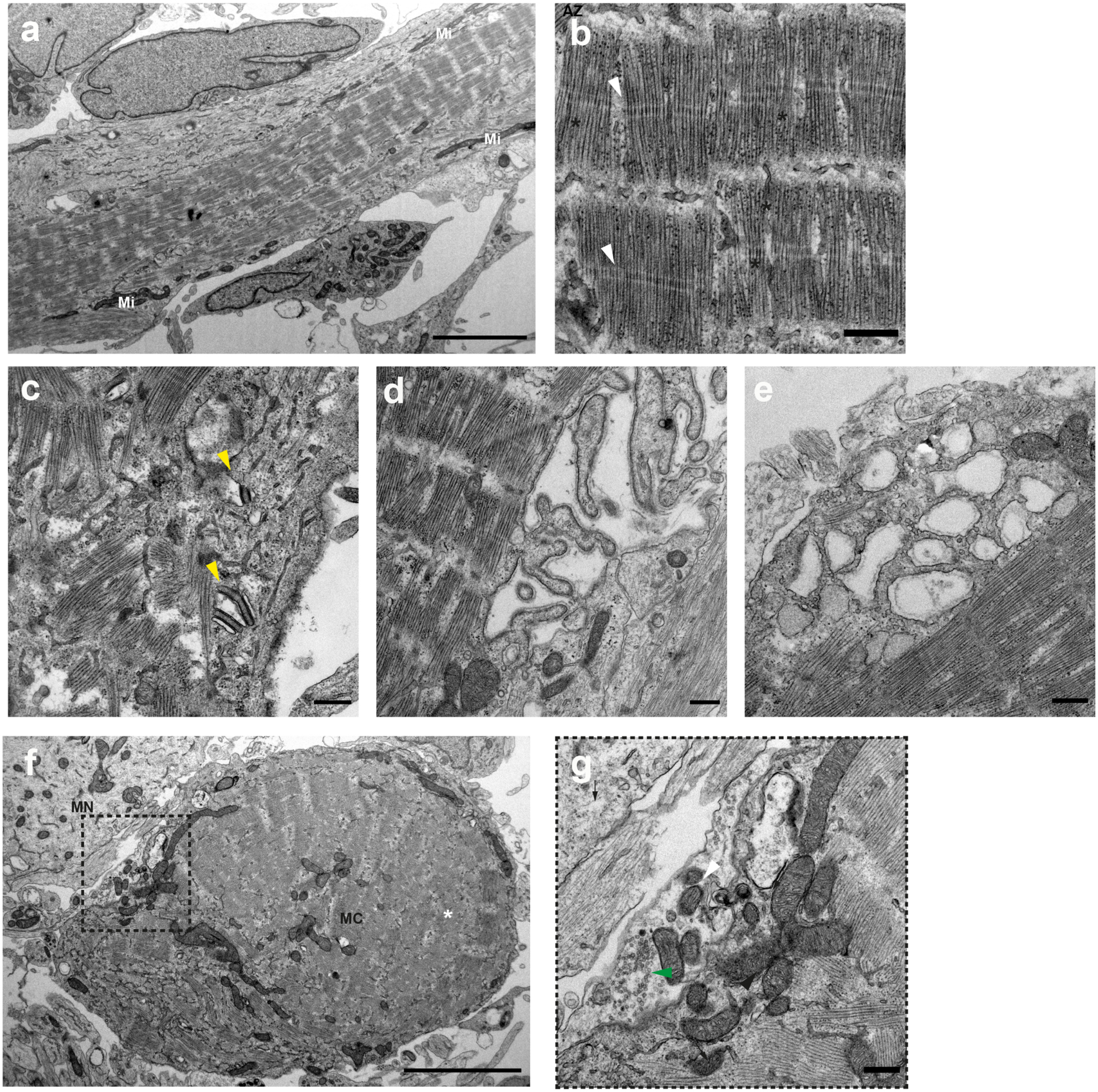

Supplementary Figure S6: Ultra-structural analysis of soNMJ cultures by electron microscopy reveals features of mature muscle fiber organization and innervation.

(a) Overview of the muscle cells of a day 100 soNMJ culture. Muscle fibers are longitudinally aligned. As known for mature muscle fibers, mitochondria are located at the periphery of the cell. Scale bar 5 µm.

(b) Sarcomeric organization in muscle cells of a day 100 soNMJ culture. Arrows point to H-zone. Scale bar 500 nm.

(c-e) soNMJ cultures show various features present in mature muscle: t-tubules (c), membrane folding with surrounding basal membrane (d), and invagination sites with many caveolae (e). Yellow arrows in (c) point to T-tubules. Scale bar 500 nm.

(f) Cross section of a muscle fiber in close association with a motor neuron. Scale bar 5 µm.

(g) Close-up of contact site: neuron with accumulated synaptic vesicles (green arrow). Scale-bar 500 nm.

soNMJ: self-organizing neuromuscular junction.

Supplementary Figure S7

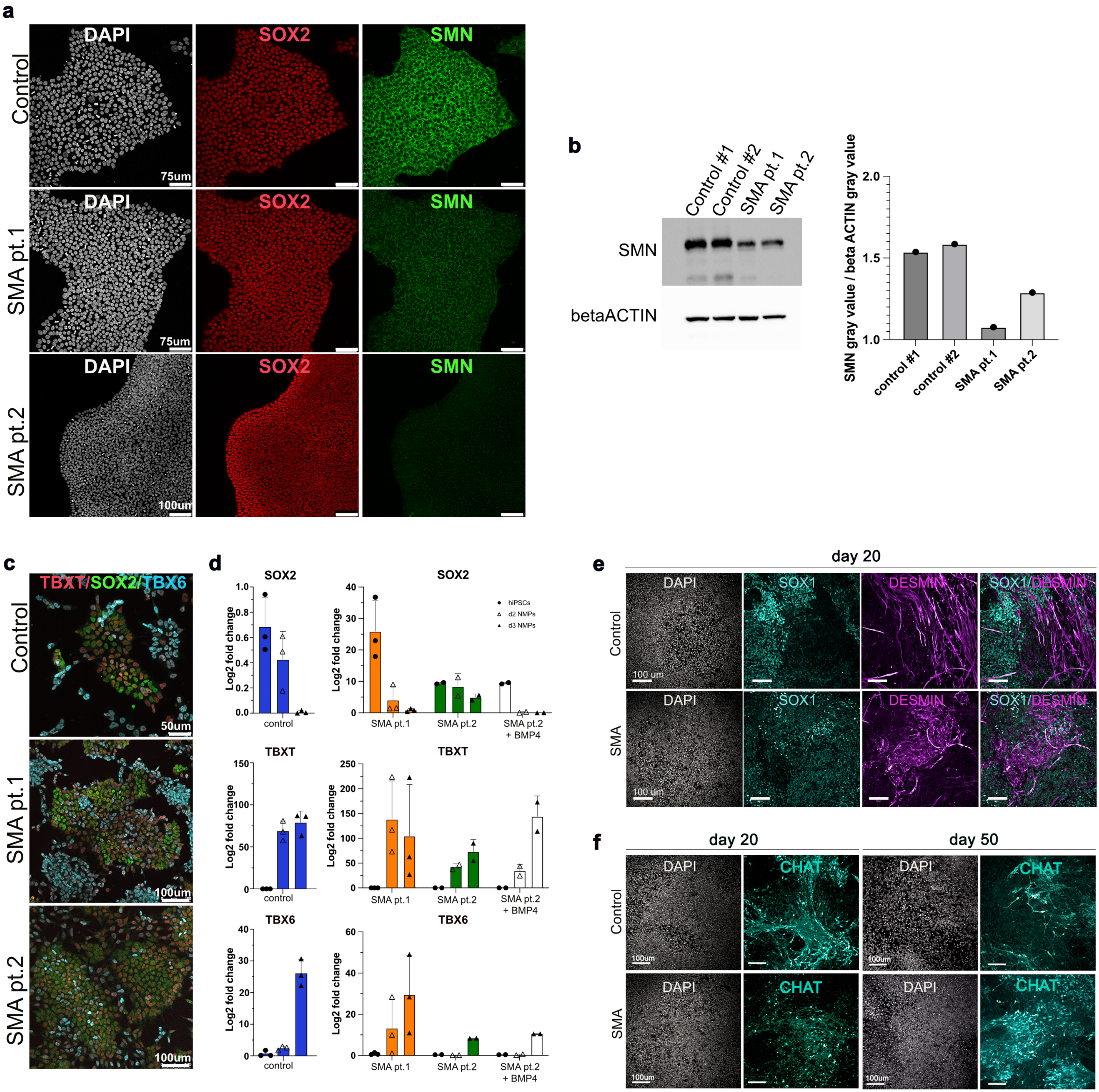

Supplementary Figure S7: SMA hPSC cells differentiate into soNMJ cultures.

(a) Immunofluorescence analysis of H1, SMApt1, and SMApt2 hPSCs showing expression of the pluripotency marker SOX2 and the reduced expression of SMN protein in both SMA type 1 human iPSCs compared to the control. Scale bar 75 and 100 µm.

(b) Western blot analysis confirms reduced SMN protein levels in both SMA type 1 iPSC lines compared to control lines. Beta-ACTIN was used for normalization.

(c) Immunofluorescence analysis of day 3 NMPs from SMApt1 and SMApt2 iPSCs showing the expression of TBXT, SOX2 and TBX6 markers. Scale bars 50 µm and 100 µm.

(d) qPCR analysis of control (shown in blue), SMApt1 (shown in orange), and SMApt2 (shown in green) iPSCs, day 2, and day 3 NMPs showing the expression of TBXT, SOX2, and TBX6 genes. SMApt2 required the addition of BMP4 to efficiently generate NMPs (shown in white). Source data are provided as a Source Data file.

(e) Immunofluorescence analysis of H1 and SMA type I soNMJ models at day 20 revealed the presence of $SOX1^+$ neural progenitors and $DESMIN^+$ myoblasts in both cell lines. Scale bar 100 µm.

(f) Immunofluorescence analysis of H1 and SMA type I soNMJ models at day 20 and 50 revealed the presence of $CHAT^+$ MNs in both cell lines. Scale bar 100 µm.

hPSCs: human pluripotent stem cells; SMA: Spinal muscular atrophy; NMPs: neuromesodermal progenitors; SMApt1: Spinal muscular atrophy patient 1; SMApt2: Spinal muscular atrophy patient 2.

Supplementary Figure S8

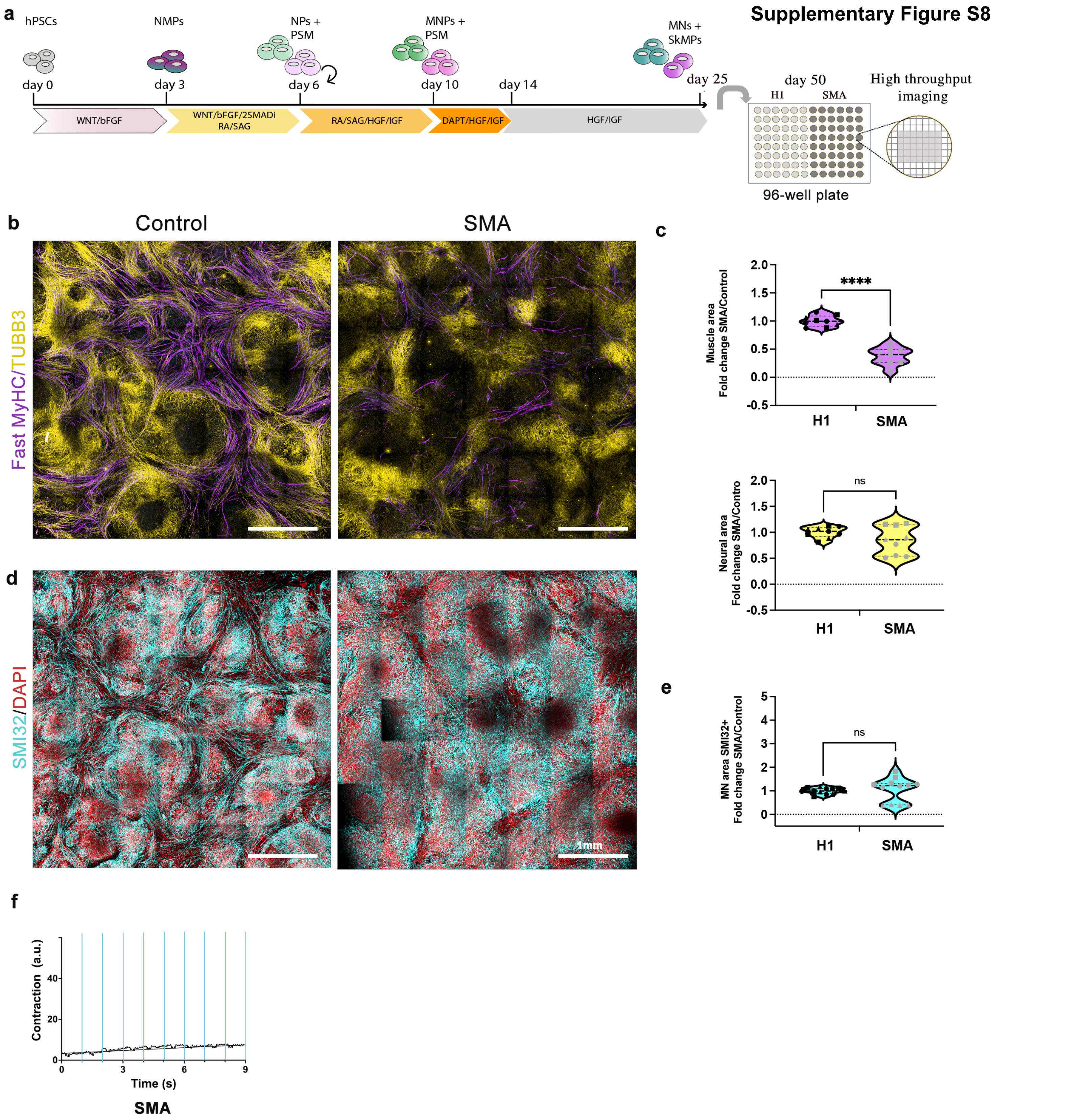

Supplementary Figure S8: High-throughput imaging and analysis of soNMJ cultures revealed impaired muscle formation in SMA human PSC-derived cultures.

(a) Differentiation strategy adapted for the high-content and high-throughput imaging of soNMJs.

(b) Immunofluorescence analysis of control and SMA soNMJ cultures at day 50 revealed reduced number of muscle fibers in SMA cultures compared to control. Antibodies against fast myosin heavy chain and beta-tubulin were used. Scale bar 1 mm.

(c) The muscle area is reduced by 50% in the SMA soNMJ model, whereas the neural area remains similar between the SMA and control soNMJ cultures. Both the muscle (Fast $MyHC^+$) and neural ($TUBB3^+$) areas were normalized to the DAPI area. The statistical tests employed included an unpaired t-test with Welch's correction (****$P \leq 0.0001$). Each dot represents measurements from an individual 96 well, and different shapes represent wells from different experiments. Source data are provided as a Source Data file.

(d) Immunofluorescence analysis of motor neurons using an antibody against neurofilament H (SMI-32) at day 50 of control and SMA soNMJ cultures. Scale bar 1 mm.

(e) The SMI-32^+ area / DAPI area is similar in the control and SMA soNMJ cultures.

(f) Optogenetic stimulation of SMA soNMJ cultures failed to induce synchronous muscle contraction.

SMA: spinal muscular atrophy; soNMJ: self-organizing neuromuscular junction.

Table S1: Summary of the number of independent experiments performed for each immunofluorescence image in the main and supplementary figures.

Figure	Selected image	Number of independent experiments
Fig 1C	H1	x3 H1, x3 H9, x3 XM001
Fig 1D	XM001	x3 H1, x3 H9, x3 XM001
Fig 2A	H1	x2 H1, x2 XM001
Fig 2A	H1	x2 H1, x3 XM001
Fig 2A	H1	x3 H1, x3 XM001
Fig 2D	H1	x3 H1, x2 XM001
Fig 2D	H1	x3 H1, x3 XM001, x1 H9
Fig 2D	H1	x3 H1, x1 XM001, x1 H9
Fig 2F	XM001	x3 H1, x 3 XM001
Fig 3B	H1	x1 H1, x1 XM001, x1 H9
Fig 3C	H1	x3 H1, x3 XM001, x3 H9
Fig 5B	H1, SMA pt1, SMA pt2	x3 H1, x3 SMApt1, x3 SMA pt2
Fig 5D	H1, SMA pt1, SMA pt2	x3 H1, x3 SMApt1, x3 SMA pt2
Suppl Fig 1	H1	x1 H1
Suppl Fig 2B	H1	x3 H1, x3 H9
Suppl Fig 3A	H1	x1 H1
Suppl Fig 4A	H1	x3 H1, x3 XM001, x3 H9
Suppl Fig 4A	XM001	x3 H1, x3 XM001
Suppl Fig 4D	H1	x3 H1, x3 XM001
Suppl Fig 5B	H1	x3 H1, x3 XM001
Suppl Fig 5E	H1	x3 H1, x3 XM001
Suppl Fig 5F	H1	x3 H1, x3 XM001
Suppl Fig 7A	H1, SMA pt1, SMA pt2	x3 H1, x2 SMApt1, x2 SMA pt2
Suppl Fig 7C	H1, SMA pt1, SMA pt2	x3 H1, x2 SMApt1, x2 SMA pt2
Suppl Fig 7E	H1, SMA pt1	x1 H1, x 1SMA pt1
Suppl Fig 7F	H1, SMA pt1	x1 H1, x 1SMA pt1
Suppl Fig 8B	H1, SMA pt1	x3 H1, x3 SMA pt1
Suppl Fig 8D	H1, SMA pt1	x3 H1, x3 SMA pt1

Table S2: List of Antibodies

Antibody	Source	Identifier	Dilution
Goat anti-TBX6	R&D	AF4744	1:500
Goat anti-ChAT	Millipore	AB144P	1:250
Goat anti-SOX1	R&D	AF3369	1:500
Goat anti-T/BRA	R&D	NL2085R	1:250
Rabbit anti-SOX2	Millipore	AB5603	1:500
Rabbit anti-TUBIII / TUJ1	Biozol	GTX129913-25	1:1000
Rabbit anti-LAMININ	Abcam	AB11575	1:500
Rabbit anti-S100β	Abcam	AB52642	1:500
Rabbit anti-GFAP	Sigma Aldrich	SAB5600060	1:500
Rabbit anti-DESMIN	Abcam	AB15200	1:500
Rabbit anti-OLIG2	Millipore	AB9610	1:500
Mouse anti-PAX3	DSHB	AB_528426	1:50
Mouse anti-FAST MYOSIN HEAVY CHAIN	Sigma Aldrich	M1570	1:50
Mouse anti-PAX7	DSHB	AB_528428	1:50
Mouse anti-TITIN	DSHB	9D10	1:500
Mouse anti-ISL1/2	DSHB	39.4D5	1:50
Alexa 647 Conjugate α-bungarotoxin	Thermo Fisher	B35450	1:100
Mouse anti-LHX1,2	DSHB	4F2	1:50
Sheep anti-CHX10	Abcam	AB16141	1:500
Rabbit anti-PAX2	Biozol	BLD-901001	1:50

Rabbit anti-MYF5	Santa Cruz	SC 302	1:500
Rabbit anti- LHX3	Abcam	ab14555	1:500
Mouse anti-SMN	BD Transduction	610646	1:1000
Mouse anti-HB9	DSHB	815C10	1:50
Mouse anti-TUBB3 / TUJ1	Covance	MMS-435P	1:1000
Rabbit anti-HOXC6	ThermoFisher	PA-5-41479-20	1:500
Rabbit anti-FOXP1	Millipore	ABE 68	1:500
HRP anti-mouse IgG	Sigma	A2554	1:10000
Mouse Anti-β-Actin	Sigma	A2228	1:20000
Donkey anti-mouse, Alexa fluor 488	Invitrogen	A21201	1:1000
Donkey anti-rabbit, Alexa fluor 568	Invitrogen	A10042	1:1000
Donkey anti-Goat, Alexa Fluor 633	Invitrogen	A-21082	1:1000
Donkey anti-mouse, Alexa fluor 568	Invitrogen	A10037	1:1000
Donkey anti-rabbit, Alexa fluor 488	Invitrogen	A21206	1:1000
Donkey anti-Goat, Alexa Fluor 647	Invitrogen	A21447	1:1000
Donkey anti-Sheep, Alexa Fluor 488	Invitrogen	A21447	1:1000

Table S3: List of human qPCR primers

Gene	Seqeuence 5'-3'
BRACHYURY F	TGCTTCCCTGAGACCCAGTT
BRACHYURY R	GATCACTTCTTTCCTTTGCATCA
SOX2 F	TGGACAGTTACGCGCACAT
SOX2 R	CGAGTAGGACATGCTGTAGGT
TBX6 F	GGCCACCTGATCCTGCACTC
TBX6 R	GGCAAAGGGATTGGCTGCAA
SOX1 F	GTCATGTCCGAGGCCGAGAA
SOX1 R	GAGCAGCGTCTTGGTCTTGC
PAX6 F	GTACTGAATGACTCAACTGCTCGG
PAX6 R	CTTTAGAAGGAAGCGACACTCTGC
OTX2 F	CAAAGTGAGACCTGCCAAAAAGA
OTX2 R	TGGACAAGGGATCTGACAGTG
CDX2 F	GTCCCAGAGCCCTTGAGTCC
CDX2 R	GCAGAGTCCACGCTCCTCAT
HOXB1 F	GGTCAAGATTTGGTTCCAGAACCG
HOXB1 R	ATTGGTGGCTAGGTTCAGTTCAGG
NKX1.2 F	AGCTGGTGGCCTTGGAGAAC
NKX1.2 R	AGAGACAGCGCGAGGTTCAG
MEOX1 F	TCTGAGCGCCAGGTCAAAG
MEOX1 R	CTGAACTTGGAGAGGCTGTGG
MYF5 F	ATGGCATGCCCGAATGTAAC
MYF5 R	GGTGATCCGGTCCACTATGT
MYOD1 F	GACGGCATGATGGACTACAG
MYOD1 R	GATGCTGGACAGGCAGTCT
MSGN1 F	AGGGCTTTAGTGCTCCTGTT
MSGN1 R	AACTTAGCCTGTCTGCCTGT
FOXC1 F	TTCGAGTCACAGAGGATCGG
FOXC1 R	TAGTTCGGCTTTGAGGGTGT
FOXC2 F	ACAGCTACATCGCGCTCATCACCAT
FOXC2 R	ATGCTGTTCTGCCAGCCCTGCTTGTT
OLIG2 F	CCCACCGACTCATCTTTCCT
OLIG2 R	GCAAACAGCTTAGCATTGCG
FOXP1 F	CAAAGAACGCCTGCAAGCCATG

FOXP1 R	GGAGTATGAGGTAAGCTCTGTGG
CHAT F	TGAGTACTGGCTGAATGACATG
CHAT R	AGTACACCAGAGATGAGGCT
ISL1 F	AGATTATATCAGGTTGTACGGGA
ISL1 R	ACACAGCGGAAACACTCGAT
