# Supplementary material for: Efficient generation of a self-organizing neuromuscular junction model from human pluripotent stem cells
Source: Nat Commun. 2023 Dec 19;14:8043. doi: 10.1038/s41467-023-43781-3 (PMC10730704; doi:10.1038/s41467-023-43781-3)
Supplement: Supplementary file 3 — Description of Additional Supplementary Files [file 41467_2023_43781_MOESM3_ESM.pdf]

Description of Additional Supplementary Files

File Name: Supplementary Movie 1
Description: 3D reconstruction of the soNMJ model (Related to Fig, 3c).

File Name: Supplementary Movie 2
Description: Contraction recordings of day 50 and day 75 soNMJ cultures and the response to pharmacological treatments (Related to Fig. 3e).

File Name: Supplementary Movie 3
Description: Calcium imaging of the soNMJ model and the response to curare (Related to Fig. 4a).

File Name: Supplementary Movie 4
Description: Optogenetic analysis of day 75 soNMJ model and the response to curare (Related to Fig. 4f).

File Name: Supplementary Movie 5
Description: SMA soNMJ contraction and the response to pharmacological treatments (Related to Fig. 5f).

File Name: Supplementary Movie 6
Description: 3D reconstruction of the PAX7/LAMININ immunofluorescence image at day 100 (Related to Supplementary Fig. 5e).

File Name: Supplementary Movie 7
Description: 3D reconstruction of a D100 soNMJ confocal image showing the presence of terminal Schwann cells (S100β+ ; green), spinal neurons (TUBB3+ ; magenta) and NMJs (αBTX; cyan). (Related to Supplementary Fig. 5f).

File Name: Supplementary Movie 8
Description: SMA optogenetic analysis of day 50 SMA soNMJ culture (Related to Supplementary Fig. 8f).
